# Epithelioid inflammatory myofibroblastic sarcoma: the youngest case reported

**DOI:** 10.4322/acr.2021.288

**Published:** 2021-05-25

**Authors:** Sajida Batool, Arvind Ahuja, Devender Singh Chauhan, Minakshi Bhardwaj, Atul Kumar Meena

**Affiliations:** 1 Atal Bihari Vajpayee Institute of Medical Sciences, Dr Ram Manohar Lohia Hospital, Department of Pathology, New Delhi, India; 2 Atal Bihari Vajpayee Institute of Medical Sciences, Dr Ram Manohar Lohia Hospital, Department of Pediatric Surgery, New Delhi, India

**Keywords:** Sarcoma, Epithelioid Cells, Intestine, Small, Mesentery, Anaplastic Lymphoma Kinase

## Abstract

Epithelioid inflammatory myofibroblastic sarcoma (EIMS) is a rare variant of the inflammatory myofibroblastic tumor. It has an aggressive clinical course and a high rate of recurrence. EIMS primarily affects children and young adults. Hereby, we report this entity in a 4-month-old infant who presented with an abdominal mass. Imaging studies revealed a large hypodense mesentery-based lesion involving the right half and mid-region of the abdomen. The mass with an attached segment of the small bowel was excised *in toto*. Grossly, a large encapsulated tumor was identified arising from the mesentery of the small bowel. The histological examination showed a tumor consisting of epithelioid to spindle cells loosely arranged in a myxoid background with numerous blood vessels and lymphoplasmacytic inflammatory infiltrate. On immunohistochemistry, the tumor cells showed positivity for ALK1 (nuclear), desmin, SMA, CD68, and focal positivity for CD30. A final diagnosis of EIMS of the small intestine was rendered. To the best of our knowledge, this case is the youngest reported case in literature.

## INTRODUCTION

Inflammatory myofibroblastic tumor (IMT) is a mesenchymal neoplasm with borderline malignant potential. It often affects children and young adults with the mean age at diagnosis of 10 years. It mostly arises in the mesentery, omentum, and abdominal soft tissue, followed by lung, mediastinum, and head and neck.^1^ Histologically, it is a distinctive neoplasm composed of spindled myofibroblastic cells, and inflammatory infiltrate in an edematous myxoid or hyalinized stroma with abundant blood vessels. Epithelioid inflammatory myofibroblastic sarcoma (EIMS) is a rare and recently defined variant of IMT.^2^ It is often intra-abdominal and clinicopathologically different from conventional IMT in that it is more aggressive with a higher rate of recurrence and poorer prognosis.^3^ It is mainly composed of plump round to epithelioid or histiocytoid tumor cells with a minor spindle cell component. Cells are arranged in clusters or non-cohesive sheets within a stroma, which is frequently myxoid. Necrosis is uncommon, and mitosis is variable. Most EIMS express ALK and desmin with variable smooth muscle actin and CD30 positivity. Focal CD68 positivity is seen in histiocytic appearing cells.^1^ We report this rare entity in a four-month-old infant, which is the youngest reported case in literature.

## CASE REPORT

**Figure 1:**
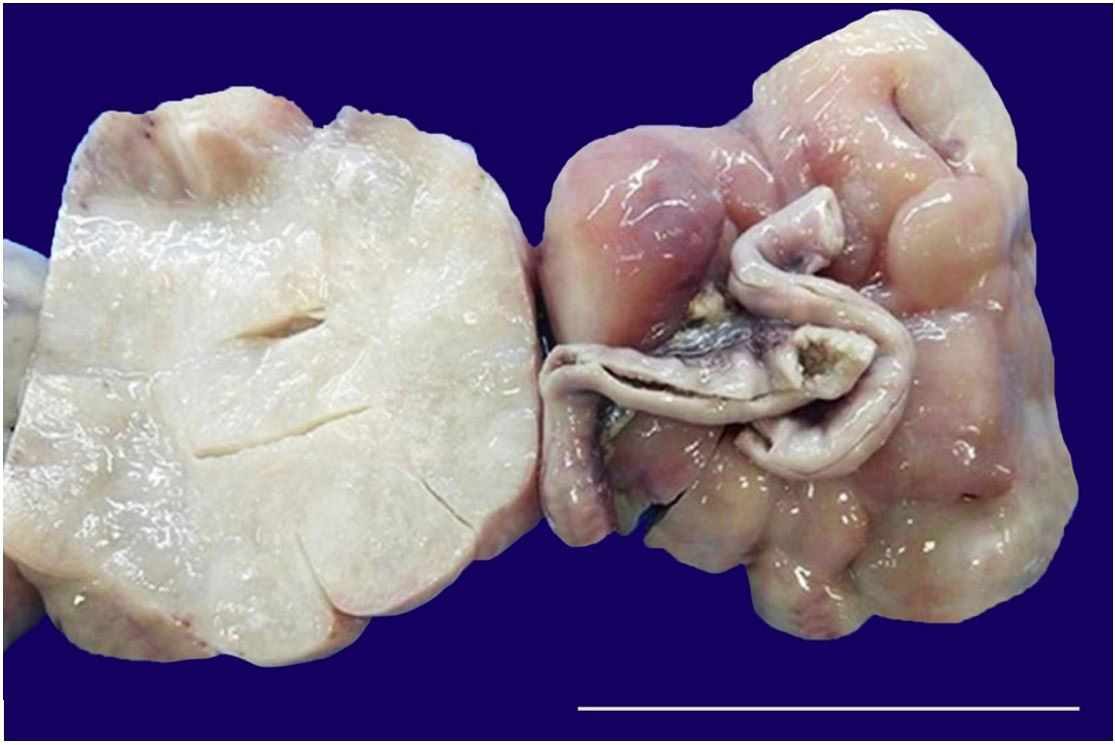
Gross examination of the tumor showing a mass with attached ileal segment and its cut surface (scale bar = 9 cm).

A 4-month-old female patient presented with a rapidly growing abdominal lump. On the abdominal examination, a large non-tender mass with ill-defined borders and side-to-side mobility was palpable in the right lumbar and umbilical region. The abdominal contrast-enhanced computed tomography (CECT) revealed a large hypodense abdominal lesion (11.4x11x8cm) involving the right half and mid-region, displacing the bowel loops posterolaterally. However, tumor invasion was not seen. No evidence of septations or solid enhancement was seen within the lesion. Clinically, the diagnosis of mesenteric cyst or teratoma was considered. The infant was operated on, and an encapsulated irregular mass attached to a small bowel segment was excised *in toto*, along with mesenteric lymph nodes. On gross examination, a large encapsulated tumor (12x11x7cm) was identified arising from the mesentery. The external surface of the mass was encapsulated and lobulated. The cut section was greyish-white and glistening with a myxoid appearance and soft to firm in consistency (Figure 1).

**Figure 2:**
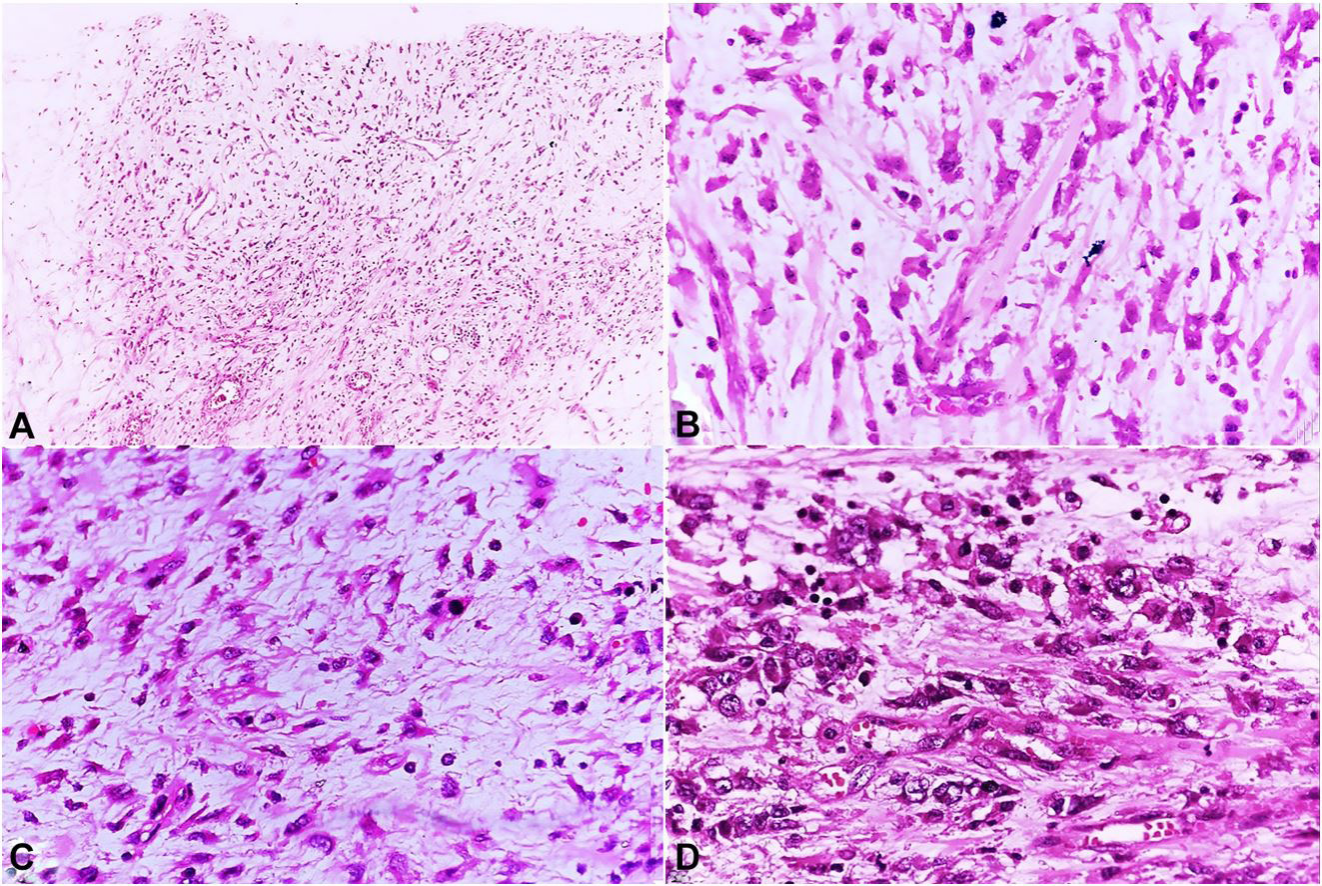
Histopathological examination of the tumor. **A** – Moderately cellular tumor with intervening small blood vessel (H&E, x100); **B** – Tumor cells with moderate eosinophilic cytoplasm and oval to spindle nuclei (H&E, x200); **C** – Myxoid area along with mild lymphoplasmacytic inflammatory infiltrate (H&E, x200); **D** – Epithelioid to round cell morphology of tumor cells with conspicuous nucleoli and few binucleated forms (H&E, x400).

**Figure 3:**
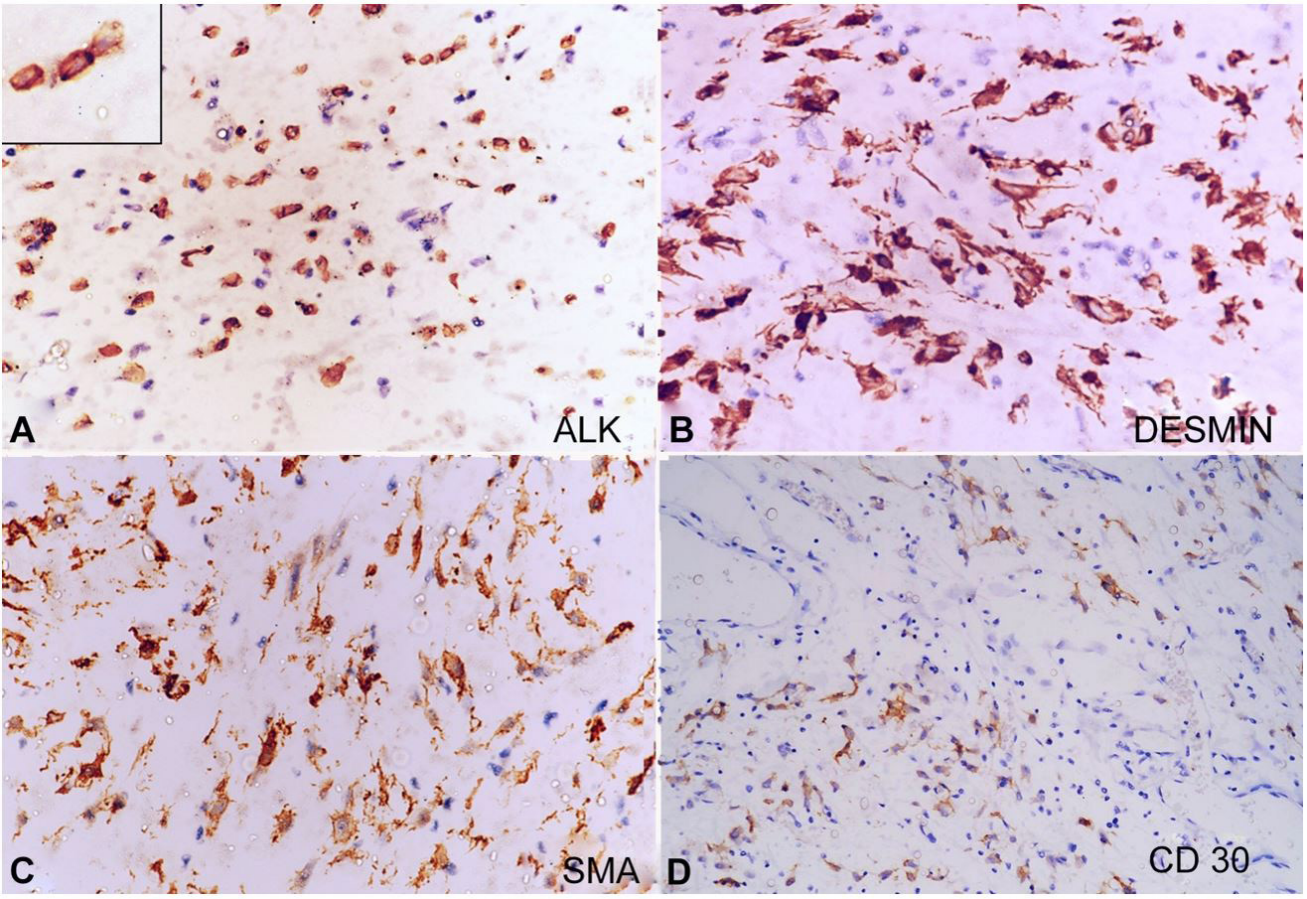
Immunohistochemistry. **A** – Tumor cells showing ALK nuclear membranous positivity (inset) (x400); **B** – Diffuse desmin positivity (x400); **C** – SMA positivity (x400); **D** – Focal CD30 positivity (x200).

On the histopathological examination, the tumor consisted of epithelioid to spindle cells loosely arranged in a myxoid background with numerous blood vessels (Figure 2).The tumor cells had moderate eosinophilic cytoplasm, oval to spindle nuclei, and conspicuous nucleoli. Epithelioid to round cell morphology was also noted in many areas with few binucleated cells. Variable mitosis (4-5/10hpf) was noted. Necrosis was not seen. There was a small amount of spindle cell component comprising about 15-20% of the tumor and was mostly scattered all around the tumor. There was mild to moderate chronic inflammatory infiltrate within the tumor, mainly comprising mature lymphocytes with scattered plasma cells. A large panel of IHC was applied, and the tumor cells showed positivity for ALK1 (nuclear membrane), desmin, SMA, CD68, and focal positivity for CD30 (Table 1) (Figure 3).

**Table 1:** Antibodies used for immunohistochemical (IHC) staining and their interpretation

**Result**	**IHC markers**
Positive	ALK, SMA, CD30, CD68, and Desmin
Negative	Pan CK, EMA, CD34, CD117, S100, CD99, BCL2
	MyoD1, Myogenin

ALK, anaplastic lymphoma kinase; BCL, B-cell lymphoma; CD, cluster of differentiation; CK, cytokeratin; EMA, epithelial membrane antigen; MyoD1, myoblast determination protein 1; SMA, smooth muscle actin; S100, solubility in 100%ammonium sulphate.

Based on the morphology and immunohistochemistry results, a final diagnosis of epithelioid inflammatory myofibroblastic sarcoma of the small intestine was made. Both gross and microscopic examinations from the small bowel’s attached segment were unremarkable and not involved by the tumor. Few lymph nodes were dissected out which showed reactive lymphoid hyperplasia. The immediate postoperative period was uneventful. The patient was discharged after ileo-ileal anastomosis, in a stable condition and normal passage of stool. On follow-up after 6 months, the general condition of the patient was fair, and she did not have any complaint.

## DISCUSSION

EIMS occurs mostly in children and adolescents and has a male predilection, contrary to the conventional IMT, which has a slight female predominance. The exact frequency of EIMS remains underestimated, as it has not been widely documented. Only 43 cases have been reported in the literature.^4^ ^-^ ^7^ The age of the patients ranged from 7 months to 63 years.^4^ Most of the tumors were intra-abdominal, of which 12 involved the mesentery, and out of these 12 cases, 9 involved the mesentery of the small bowel. Most cases were unifocal, with a tumor size ranging from 5 to 26 cm in its longest axis. All cases were ALK-positive on immunohistochemistry. Clinically, these patients presented with abdominal pain or masses and sometimes with ascites. Our case is the 10^th^ case of small bowel mesentery and the youngest among all the reported cases.

EIMS often displays the following common histomorphological and immunohistochemical characteristics: i) round to epithelioid tumor cells with scattered inflammatory infiltrates, ii) abundant myxoid stroma, iii) expression of ALK (nuclear membrane), and desmin (cytoplasmic, diffuse, and strong).^8^ In addition, it can also display a variable expression of SMA, CD30, CD68, and cytokeratin.^4^ EIMS may show nuclear membrane staining pattern or cytoplasmic positivity with focal perinuclear accentuation pattern of ALK.^9^ There is aberrant expression of anaplastic lymphoma kinase (ALK) protein in 50-60% of the cases due to clonal rearrangements of the *ALK* gene located on chromosome 2p23.^10^ Several ALK fusion proteins, including RNA binding protein 2 (RANBP2)-ALK, tropomyosin 3 (TPM3)-ALK, and ribosome binding protein 1 (RRBP1)-ALK are identified in EIMS and are associated with an aggressive clinical course.^11^ ^-^ ^13^

Microscopic diagnosis of EIMS can be challenging due to its unique epithelioid to round cell morphology and atypical nuclear features, which resembles many other tumors like a gastrointestinal stromal tumor (GIST), anaplastic large cell lymphoma (ALCL), and epithelioid leiomyosarcoma (ELMS), rhabdomyosarcoma (RMS), low-grade fibromyxoid sarcoma (LGFMS) and malignant mesothelioma (MM). ALCL can be difficult to distinguish from EIMS, as the rare sarcomatoid variant of ALCL can exhibit spindle cell morphology and overlapping immunostaining, including reactivity for CD30, ALK, and SMA and non-reactivity for EMA. However, strong expression of desmin and the distinctive nuclear membrane pattern of ALK staining are not observed in ALCL. The RANBP2-ALK has never been reported in ALCL either. Epithelioid GIST is positive for CD117, DOG1, CD34 while negative for ALK staining. Mutations of c-Kit and platelet-derived growth factor-α are also present in GIST. ELMS usually displays higher cellularity and pleomorphism. Also, it generally lacks an extensive myxoid background, inflammatory infiltrates, and ALK expression. The solid variant of alveolar RMS is frequently ALK-positive; however, it lacks fibrovascular stroma and forms sheets of round cells with variable rhabdomyoblastic differentiation. Myogenin and MyoD are highly specific and sensitive for its diagnosis. LGFMS shows bland spindle cells with a whorling growth pattern and arcades of curvilinear blood vessels in an admixture of collagenous and myxoid stroma. LGFMS is positive for MUC4 and EMA, focal SMA, and desmin positivity while not expressing ALK. CK5 and calretinin are positive in MM, but ALK and desmin are absent.^1^ ^,^ ^8^ Panel of IHC commonly used for differential diagnosis is depicted in Table 2.

**Table 2:** Main differential diagnoses of EIMS

**Tumor**	**Immunohistochemistry**
EIMS	ALK +, SMA +, EMA +/-, CK +/-, CD30 +, CD34 -, and Desmin +
GIST	CD117 +, DOG1 +, CD34 +, and Desmin -
ALCL	ALK +, SMA +, EMA +, CD30 +, and Desmin -
ELMS	ALK -, SMA +, EMA + and Desmin +
LGFMS	EMA +, MUC4 +, CD99, BCL2, SMA +/-, and Desmin +/-
RMS	ALK+/-, Myogenin +, MyoD1 +, Desmin +, SMA +, EMA -, CD30 -
MM	CK5 +, Calretinin +, EMA +/-, and Desmin -
Our case	ALK +, SMA +, CD30 +, CK-, EMA -, CD34 -, and Desmin +

ALCL, anaplastic large cell lymphoma; ALK, anaplastic lymphoma kinase ; BCL, B cell lymphoma; CD, cluster of differentiation; CK, cytokeratin; DOG1, discovered on GIST 1 ; EIMS, epithelioid inflammatory myofibroblastic sarcoma; EMA, epithelial membrane antigen; ELMS, epithelioid leiomyosarcoma, ; GIST, gastrointestinal stromal tumor; LGFMS, low grade fibromyxoid sarcoma; MM, malignant mesothelioma; MUC, mucin; MyoD1, myoblast determination protein 1; RMS, rhabdomyosarcoma; SMA, smooth muscle actin.

It is important to recognize EIMS as a distinct variant of IMT as patients with ALK-rearranged EIMS may benefit from targeted therapy.^3^The mainstay of treatment for IMT/EIMS is complete surgical resection when possible. However, recurrence is common. A few case reports described the combined therapy of surgery and systemic therapy with ALK inhibitors when an ALK mutation is present.^14^ The effectiveness of alternative treatment modalities like radiotherapy and chemotherapy is uncertain.

## CONCLUSION

Epithelioid inflammatory myofibroblastic sarcoma is a rare and distinct variant of IMT, which mainly consists of cells with an epithelioid or round cell morphology, with a high potential of recurrence and a poorer prognosis. Only 43 cases have been reported in the literature, of which only 9 were located in the mesentery of the small bowel. This is the 10^th^ case reported in the mesentery of small bowel and youngest among all the reported cases to the best of our knowledge
